# Genome-Wide Identification and Expression Profiles of Nuclear Factor Y A Transcription Factors in Blueberry Under Abiotic Stress

**DOI:** 10.3390/ijms252312832

**Published:** 2024-11-28

**Authors:** Xiuyue Xu, Hong Su, Shuwei Sun, Jing Sun, Xiang Zhang, Jiajie Yu

**Affiliations:** 1 Forestry College, Northeast Forestry University, Harbin 150040, China; 15663593162@163.com; 2 School of Agriculture, Liaodong University, Dandong 118003, China; suhong19871014@126.com (H.S.); sswby@163.com (S.S.); sunjing19871127@126.com (J.S.); 3 State Key Laboratory of Tree Genetics and Breeding, Northeast Forestry University, Harbin 150040, China

**Keywords:** *Vaccinium corymbosum*, NF-YA transcription factors, abiotic stress, bioinformatics analysis

## Abstract

Nuclear Factor Y A (NF-YA) transcription factors are widely involved in multiple plant biological processes, such as embryogenesis, abscisic acid signaling, and abiotic stress response. This study presents a comprehensive genome-wide identification and expression profiling of NF-YA transcription factors in blueberry (*Vaccinium corymbosum*), an important economic crop with good adaptability, under abiotic stress conditions. Given the economic significance and health benefits of blueberries, understanding their responses to environmental stresses, such as salt, drought, and temperature extremes, is crucial. A total of 24 NF-YA transcription factors were identified through bioinformatics analyses, including sequence alignment, phylogenetic tree construction, and conserved motif analysis. The expression patterns of these *NF-YA* genes were evaluated in various tissues (roots, stems, and leaves) and under different stress treatments (abscisic acid, salt, and cold) using quantitative real-time PCR (qRT-PCR). The results indicated that most *VcNF-YA* genes exhibited higher expression levels in stems and leaves compared to roots. Most *VcNF-YA*s were responsive to the stress treatment. Furthermore, *cis*-acting element analysis revealed that the promoters of *VcNF-YA*s were enriched with elements responsive to abiotic stress, suggesting their pivotal role in stress adaptation. This research unveils the expressional responses of NF-YA transcription factors in blueberry upon abiotic stresses and lays the groundwork for future studies on improving crop adaptation.

## 1. Introduction

Plant growth is affected by various abiotic stresses, such as salt, drought, high or low temperature, and heavy metal stress. Facing unpredictable or continuous adverse conditions, they have gradually developed multiple strategies for survival during long-term evolution, one of which refers to transcriptional regulation. Transcription factors, as a kind of protein, play a key and central role in the process of transcriptional regulation by specifically binding to the *cis*-acting elements in the promoters of downstream genes and thereby activating or repressing their transcription [1].

*NF-Y*, also known as *CBF* (*CCAAT*-binding factor) or HAP (hem activator protein), is a heterotrimeric complex transcription factor family widely distributed in yeast, mammals, plants, and other eukaryotes. It consists of three highly conserved subfamilies: NF-YA (HAP2 or CBF-B), NF-YB (HAP3 or CBF-A), and NF-YC (HAP5 or CBF-C) [2]. NF-Y can regulate the expression of downstream target genes by specifically binding to CCAAT boxes [3]. The function of NF-Y transcription factors must be achieved by forming heterodimers or heterotrimers rather than by a single subunit. In general, NF-YB and NF-YC interact in the cytoplasm to form a dimeric complex, which then binds to NF-YA to form a mature heterotrimeric complex. Subsequently, the heterotrimeric complex binds to the promoters of downstream genes to regulate their expression. Protein structure analysis shows that there are two conserved alpha-helical domains in the core region of the NF-YA subunit. The 20-amino-acid alpha helix A1 is located at the N-terminus of the core region and plays a key role in interactions with NF-YB and NF-YC subunits. The 21-amino-acid alpha helix A2 is located at the C-terminus, providing sequence specificity for recognizing and binding to CCAAT *cis*-acting elements [4,5]. The A subunit of NF-Y is typically located in the nucleus, and most NF-YA proteins can bind to CCAAT *cis*-acting elements in the promoter region of target genes, but with different affinities [3,4,5,6].

The preliminary reports of *NF-Y* gene in plants can be traced back to the 1990s [2,7,8,9]. In the past few decades, many studies have elucidated the biological functions of individual NF-Y subunits in *Arabidopsis* and other plant species, indicating their important roles in gamete development, embryogenesis, seed development, flowering time regulation, primary root elongation, abscisic acid signaling, drought resistance, endoplasmic reticulum stress response, hypocotyl elongation, etc. [4,10,11]. Research has found that NF-YA transcription factors are involved in the regulation of seed germination and dormancy. In rice (*Oryza sativa*), there were distinct differences in the expression levels of certain *NF-YA* genes between seed dormancy and germination periods. The low expression levels of *NF-YA*s were associated with seed dormancy release and germination initiation, which may be achieved by regulating the genes related to hormone metabolism, such as abscisic acid and gibberellin [12,13]. The NF-YA transcription factors also play an indispensable role in root development. In *Arabidopsis*, mutations in specific *NF-YA* genes led to abnormalities in root structure, such as hindered main root growth and reduced number of lateral roots. Further research has proven that NF-YA shaped root structure by regulating genes related to root cell division and differentiation [14,15,16]. Studies in various plants have shown that NF-YA interacted with other flowering regulatory factors, integrated environmental signals such as photoperiod and temperature, regulated the expression of flowering-related genes, and thus affected flowering time [17,18,19,20,21]. NF-YA transcription factors were responsive to drought stress as well. For instance, some *NF-YA* genes in wheat (*Tritium aestivum*) were upregulated upon drought stress. These upregulated NF-YA transcription factors activated the expression of a series of drought-responsive genes, including those encoding aquaporins, osmoregulatory synthase, etc., and thereby improved the plant drought resistance [22,23,24,25,26]. NF-YA transcription factors are also involved in plant response to salt stress. In soybean (*Glycine max*), NF-YA transcription factor regulated the expression of ion transport protein genes, which helped plants maintain intracellular ion balance and alleviated the toxic effects of salt ions on plant cells [27,28,29,30]. NF-YA transcription factors are also involved in plant adaptation to high and low temperature [25,31]. In tomato (*Solanum lycopersicum*), changes in the *NF-YA* gene expression under low-temperature stress affected the plant cold resistance, possibly by regulating the expression of cold-responsive genes and altering the fluidity of cell membranes [32,33].

To date, NF-YA family members have been identified and characterized in many plant species. The numbers of *NF-YA* members identified from different plant species vary greatly, with a maximum of 117 members and a minimum of 5 members. Among monocotyledonous plants, 5, 8, 22, 11, 22, and 36 NF-Y family genes were identified in *Aegilops tauschii*, *Ananas comosus*, *Hordeum vulgare*, *Oryza brachyantha*, *Triticum aestivum*, and *Zea mays*, respectively. In dicotyledonous plants, 21, 18, 10, 117, 9, 57, 37, 14, 11, 30, and 49 *NF-YAs* were identified in *Arabidopsis thaliana*, *Nicotiana benthamiana*, *Solanum lycopersicum*, *Glycine max*, *Populus euphratica*, *Populus trichocarpa*, *Salix purpurea*, *Citrus Clementina*, *Eucalyptus grandis*, *Gossypium hirsutum*, and *Brassica oleracea*, respectively [34]. Blueberry, a small-berry perennial plant in the *Vaccinium* spp. genus in the *Ericaceae* family, is rich in anthocyanidins, flavonoids, polyphenols, and antioxidants with significant medicinal and health benefits. It is of great economic and ecological value. As a shrub species with shallow roots, blueberry encounter challenges from multiple abiotic stresses like salt, alkali, and drought stress [35,36]. With the annotation of blueberry (*Vaccinium corymbosum*) genome [37], it is of urgent necessity to identify the NF-YA transcription factors in this important crop. In this study, a total of 24 NF-YA transcription factors were identified in *V*. *corymbosum* through the whole genome. We analyzed their protein sequences, chromosome locations, evolutionary relationships, basic physiochemical properties, and gene structures. The expression patterns of these genes in different plant tissues upon salt, ABA, and cold treatment were also investigated. This study provides a reference for future research on NF-YA transcription factors and plant stress response.

## 2. Results

### 2.1. Identification and Sequence Analysis of NF-YA Transcription Factors in Blueberry

The *V. corymbosum* cv. Duke, v1 genome database was searched by BlastP with the AtNF-YAs as query sequences. After Pfam analysis, HMMER prediction, and two conserved domain screenings, a total of 24 VcNF-YAs were identified (Table 1). They were named VcNF-YA1-24 according to their location order on scaffolds. The physicochemical properties of the 24 putative VcNF-YA transcription factors were analyzed. The sequence length of these proteins ranged from 106 to 343 amino acids (aa). Physiochemical properties, such as molecular weight, isoelectric point, aromaticity, instability index, aliphatic index, and GRAVY, were listed in Table 1. VcNF-YA11 and VcNF-YA14 had lower instability index (<50) than other family members. VcNF-YA11 had a positive GRAVY value, while the other members had negative ones, which indicates VcNF-YA11 is the only hydrophobic protein of all the members.

**Figure 1:**
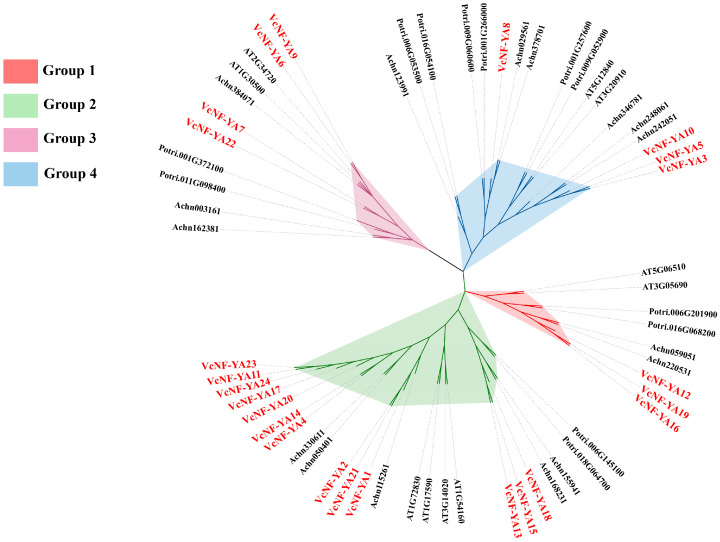
Phylogenetic relationships of NF-YA proteins from *V. corymbosum*, *A. chinensis*, *A. thaliana*, and *P. trichocarpa*. Red polygon indicates Group 1; green polygon indicates Group 2; purple polygon indicates Group 3; blue polygon indicates Group 4.

The phylogenetic tree of the NF-YAs in *V. corymbosum*, *Actinidia chinensis*, *Arabidopsis thaliana*, and *Populus trichocarpa* was constructed (Figure 1). VcNF-YAs were divided into four groups and exhibited more homology with the *NF-YA* genes in *A. chinensis*, indicating that the family members were highly conserved in the evolution process. VcNF-YA12, 16, and 19 were closely related to AT5G06510 and AT3G05690, which were clustered in Group 1. VcNF-YA6, 7, 9, and 22 were classified in Group 3, which were closely related to AT1G30500 and AT2G34720. VcNF-YA3, 5, 8, and 10 were closely related to AT3G20910 and AT5G12840, which were clustered in Group 4. Other members were classified in Group 2.

**Figure 2:**
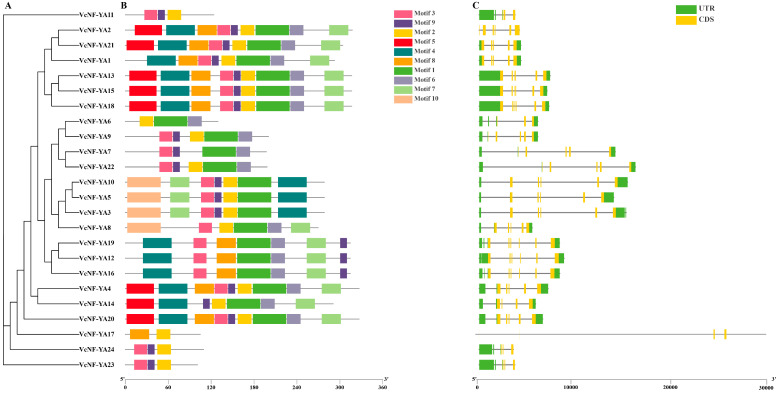
Phylogenetic relationship, exon-intron gene structure, and structure of conserved protein motifs in VcNF-YA transcription factors. (**A**) Phylogenetic tree was constructed based on the full-length sequence of the VcNF-YAs protein. (**B**) Motif composition in VcNF-YAs protein. The pattern of Motif 1–10 is displayed with boxes in different colors. Motifs in the amino acid sequence of VcNF-YAs were predicted using online MEME tool. (**C**) Exon-intron structure of the VcNF-YA transcription factors. Yellow boxes indicate exons. Green boxes indicate untranslated 5′- and 3′-regions. Black lines indicate introns.

The results of motif and gene structure analyses were visualized with the aid of the phylogenetic tree of VcNF-YAs generated based on the full-length protein sequences of VcNF-YAs (Figure 2A). The phylogenetic relationships of VcNF-YAs shown here were consistent with the result in Figure 1. The gene structure (exon-intron) analysis (Figure 2C) showed that only *VcNF-YA17* contained a long intron, with a length longer than 25 kb. Except for *VcNF-YA2* and *VcNF-YA17*, which contained neither 5′-regions nor 3′-regions, all members contain both 5′-regions and 3′-regions. Based on the results of the motif analysis by using MEME, schematic representations of the structures of all VcNF-YA proteins were constructed (Figure 2B), and the number of conserved motifs obtained was set to 10. Motif 2, 3, and 9 existed in almost all members. Transcription factors in the same class tended to have similar motif structures. For example, VcNF-YA13, 15, and 18 had roughly the same motif structures (Motif 5), while VcNF-YA12, 16, and 19 did not contain this motif. The 10 motif sequences, width, E-value, and conserved domain were labeled in Table S1.

### 2.2. Multiple Sequence Alignment Analysis of the 24 VcNF-YAs

**Figure 3:**
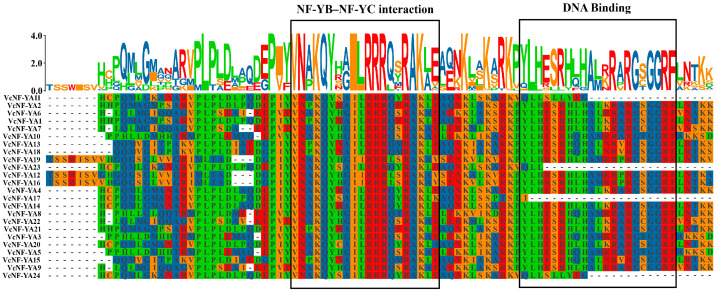
Multiple sequence alignment of VcNF-YAs. The amplified colored letters above protein sequences indicate motif. Different colors of letters represent different amino acids.

It was reported in previous studies that NF-YA protein has two conserved domains, including NF-YB–NF-YC interaction and DNA binding domain. In this study, a multiple sequence alignment of the 24 VcNF-YAs was performed. All of these conserved domains were present in the 24 VcNF-YAs (Figure 3). However, the DNA binding domains of VcNF-YA11, 17, 23, and 24 were not complete.

### 2.3. Chromosome Location and Intraspecies Collinearity of VcNF-YAs

**Figure 4:**
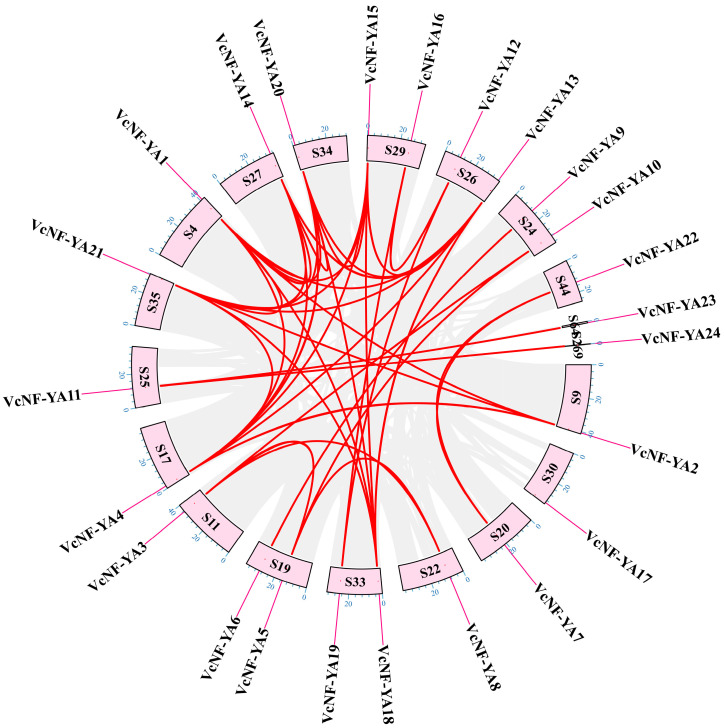
Chromosomal localization and intraspecific collinearity analysis of *VcNF-YA* genes. Pink boxes represent scaffolds; scale is shown in blue on each pink box; purple line indicates the location of the *NF-YA* genes; gray lines in the background indicate the synteny blocks within the genome; red curve denotes a pair of synteny event.

The chromosome location of the 24 *VcNF-YAs* was mapped based on the genomic information of *V. corymbosum* (Figure 4). The results showed that these 24 genes were located on three different scaffolds (S4, S9, S11, S17, S19, S20, S22, S24, S25, S26, S27, S29, S30, S33, S34, S35, S44, S64, and S269). The investigation of gene duplication is important for studying the expansion and evolution of the NF-YA genes in *V. corymbosum*. Gene duplication events play an essential role in plant evolution, containing tandem duplication and segmental duplication. Specifically, 23 *VcNF-YA* gene pairs were identified, which implied gene duplication events of *VcNF-YA*s during the evolution of *V. corymbosum* (Figure 4). The intraspecies collinearity of *NF-YA*s in *Vaccinium corymbosum* was displayed in Table S2. As shown in Table S2 and Figure 4, there were multiple collinear relationships among the VcNF-YA family members. Among them, *VcNF-YA1* and *VcNF-YA21* participated in the largest number of collinear relationships in the blueberry genome. However, *VcNF-YA17* did not have collinear relationships with other members. To better understand the evolutionary constraints on the *VcNF-YA*s, the Ka/Ks ratios of the *VcNF-YA* gene pairs were calculated. As shown in Table S3, *VcNF-YA1*, *VcNF-YA2*, and *VcNF-YA3* have no evolutionary relationships with other members. Ka represents non-synonymous replacement rate, and Ks represents synonymous replacement rate. Except for six gene pairs that underwent positive selection (Ka/Ks > 1.0), the other 131 gene pairs underwent purification selection (Ka/Ks < 1.0).

### 2.4. Interspecies Collinearity of VcNF-YAs

**Figure 5:**
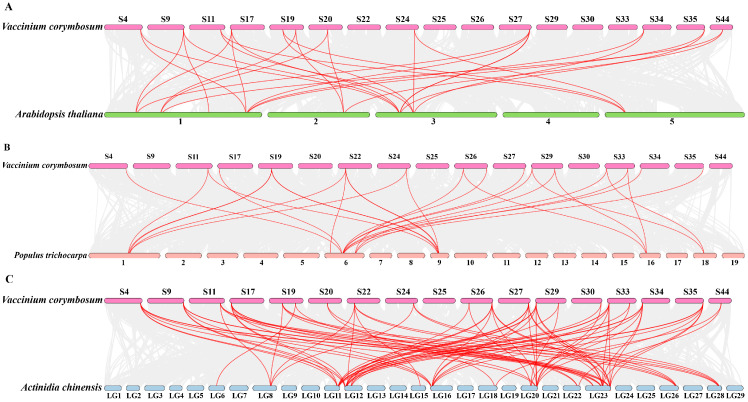
Interspecific collinearity analysis of *NF-YA*s between *V. corymbosum* and *A. thaliana* (**A**), *V. corymbosum* and *P. trichocarpa* (**B**), and *V. corymbosum* and *A. chinensis* (**C**). Grey lines in the background represent collinear blocks in *V. corymbosum* and the other three plants, respectively. Red lines represent collinear *NF-YA* gene pairs. The numbers on the chromosomes or scaffolds indicate chromosome or scaffold number.

In order to explore the duplication mechanism of *VcNF-YA* transcription factor family, we constructed comparative syntenic maps between *V.corymbosum* and *A. thaliana*, *V.corymbosum* and *A. chinensis*, and *V.corymbosum* and *P. trichocarpa*, respectively (Figure 5). The results showed that the *VcNF-YA*s had more syntenic gene pairs with the *AcNF-YA*s than with *AtNF-YA*s and *PtNF-YA*s. The number of syntenic *NF-YA* gene pairs was 28 between blueberry and *Arabidopsis* (Table S4), 28 between blueberry and poplar (Table S5), and 83 between blueberry and *A. chinensis* (Table S6). *VcNF-YA13* had the most collinear genes within the other three plants (11). *VcNF-YA1*, *2*, *3*, *8*, *19*, *20*, and *21* also had collinear genes within the other three plants. The collinear *NF-YAs* between *V. corymbosum* and *A. thaliana*, *V.corymbosum* and *P. trichocarpa*, and *V.corymbosum* and *A. chinensis* were displayed in Tables S4–S6, respectively.

### 2.5. Analysis of cis-Acting Elements in the Promoter Regions of VcNF-YAs

**Figure 6:**
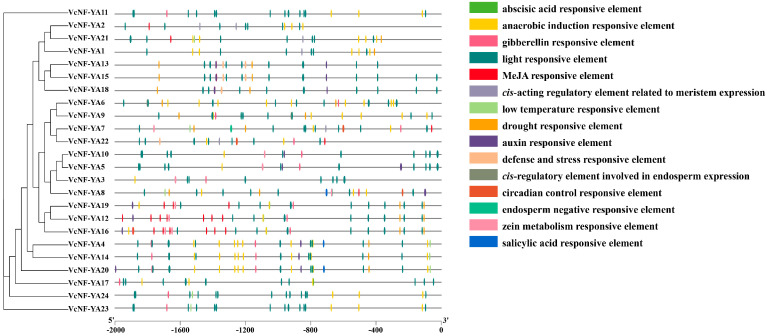
*cis*-acting elements in the promoters of *VcNF-YA*s. Different colored boxes represent different *cis*-acting elements.

A total of 15 *cis*-acting elements were identified from the 2,000 bp sequences upstreaming the CDS (coding sequence) of *VcNF-YA*s (Figure 6). These elements were mainly involved in plant hormone response, growth and development, and stress response. Plant hormone response elements included abscisic acid-responsive element, auxin-responsive element, gibberellin-responsive element, MeJA-responsive element, and salicylic acid-responsive element. Growth and development elements included zein metabolism-responsive element, light-responsive element, endosperm negative-responsive element, *cis*-regulatory element involved in endosperm expression, *cis*-acting regulatory element related to meristem expression, circadian control-responsive element, and cell cycle regulation element. Stress response elements included anaerobic induction-responsive element, defense and stress-responsive element, drought-responsive element, and low temperature-responsive element. The most frequent were light-responsive element, anaerobic induction-responsive element, MeJA-responsive element, abscisic acid-responsive element, and drought-responsive element. It showed that *VcNF-YAs* may be closely related to plant abiotic stress response. The location information of these elements were listed in Table S7.

### 2.6. Tissue-Specific Expression Analysis of VcNF-YAs

**Figure 7:**
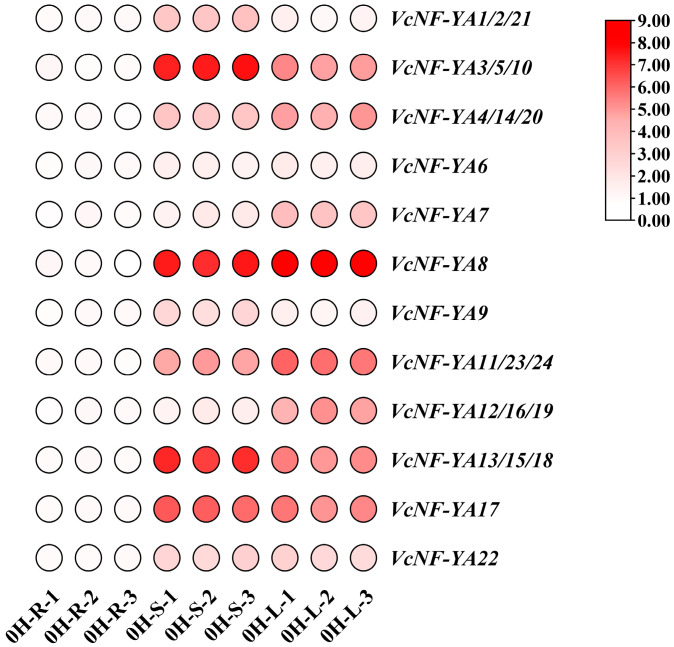
Tissue-specific expression patterns of *VcNF-YA*s. Tissue-specific expression analysis is performed with roots, stems, and leaves of *V. corymbosum* by using qRT-PCR. Internal reference gene is *GAPDH*. The expression level of each gene in roots was normalized to 1.0, calculated with 2^−ΔΔCt^ method and displayed in log_2_ (sample/control) value.

To elucidate the expression patterns of *VcNF-YA*s, the relative expression levels of these genes in roots, stems, and leaves of *V. corymbosum* were measured using qRT-PCR (Figure 7). Overall, most of these genes were expressed at higher levels in stems or leaves than in roots. Additionally, the difference degree between the relative expression levels of each gene in different tissues diverges between these genes. For instance, the relative expression level of *VcNF-YA8* in stems was 250.78 times as in roots and 1.47 times as in leaves. In contrast, *VcNF-YA22* was expressed in stems and leaves at nearly the same level, which was approximately six times as in roots. Besides, the relative expression levels of some genes, like *VcNF-YA6*, did not exhibit obvious differences between the three tissues. The related qRT-PCR data were listed in Table S8.

### 2.7. Expression Patterns of VcNF-YAs Under ABA Treatment

**Figure 8:**
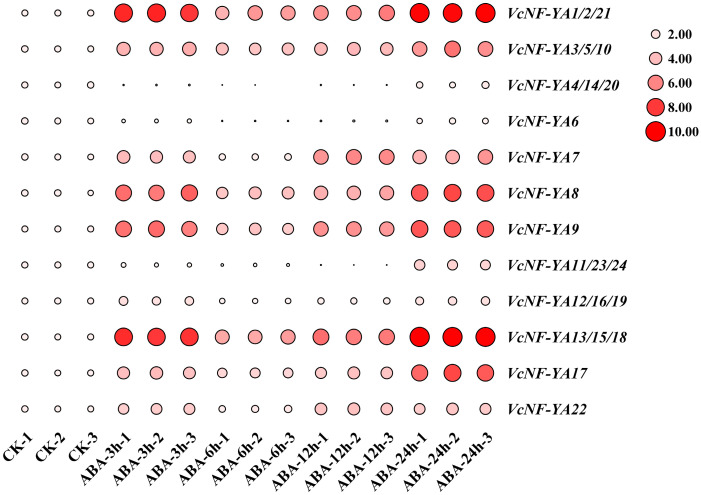
Expression patterns of *VcNF-YA*s under ABA treatment. The concentration of ABA treatment is 100 μM, and the treatment time is 0 h (CK), 3 h, 6 h, 12 h, and 24 h. The 2^−ΔΔCt^ method was used to calculate the transcription levels of *VcNF-YA*s, and the log_2_ (sample/control) value of each *VcNF-YA* was used to show relative expression levels. Expression level indicators were laid on the right side of heatmaps. Larger size and deeper red color of a circle indicate higher relative expression level.

Since the accumulation of ABA exists widely in plants upon abiotic stresses, it is necessary to investigate the response of *VcNF-YA*s to ABA treatment. The results of *cis*-acting element analysis also proved this necessity. ABA treatment was performed at different times (0 h, 3 h, 6 h, 12 h, and 24 h) on blueberry seedlings, and the relative expression levels of these genes in leaves were measured by qRT-PCR (Figure 8). The results showed that all *VcNF-YA*s were responsive to ABA treatment. The expression levels of most *VcNF-YA*s increased since the ABA treatment began, reached a peak at 3 h time point, decreased back to a certain extent thereafter, and reached a new peak at a time point of 12 h or 24 h. Exception also existed that *VcNF-YA11/23/24* were downregulated since the ABA treatment began and until 12 h and were expressed at the highest level at 24 h. All the raw data of expression level were listed in Table S9.

### 2.8. Expression Patterns of VcNF-YAs Under Salt Stress

**Figure 9:**
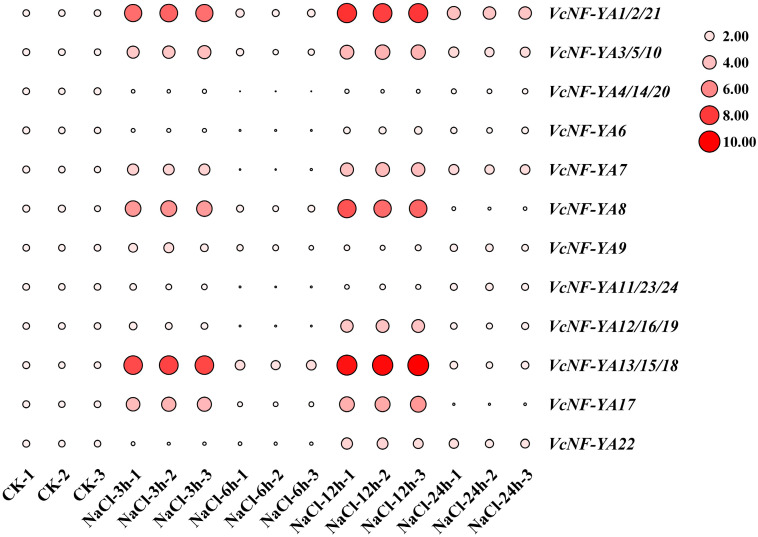
Expression patterns of *VcNF-YA*s under salt treatment. The concentration of salt treatment is 200 mM, and the treatment time is 0 h (CK), 3 h, 6 h, 12 h, and 24 h. The 2^−ΔΔCt^ method was used to calculate the transcription levels of *VcNF-YA*s, and the log_2_ (sample/control) value of each *VcNF-YA* was used to show its relative expression level. Expression level indicators were laid on the right side of heatmaps. Larger size and deeper red color of a circle indicate higher relative expression level.

Salt treatment was performed for different time periods (0 h, 3 h, 6 h, 12 h, and 24 h) on blueberry seedlings, and the relative expression levels of these genes in leaves were measured by qRT-PCR (Figure 9). The results showed that *VcNF-YA1/2/21*, *VcNF-YA3/5/10*, *VcNF-YA7*, *VcNF-YA8*, *VcNF-YA13/15/18*, and *VcNF-YA17* were upregulated since the salt treatment began and reached a peak at 3 h. After the low expression level at 6 h, these genes were upregulated to a new peak at 12 h and downregulated at 24 h. In contrast, the expression levels of *VcNF-YA4/14/20*, *VcNF-YA6*, *VcNF-YA11/23/24*, and *VcNF-YA12/16/19* showed completely different trends. *VcNF-YA22* was downregulated since the salt treatment began, upregulated at 12 h, and downregulated thereafter. *VcNF-YA9* was not as responsive to salt treatment as other members. All the raw data of relative expression level were listed in Table S10.

### 2.9. Expression Patterns of VcNF-YAs Under Cold Stress

**Figure 10:**
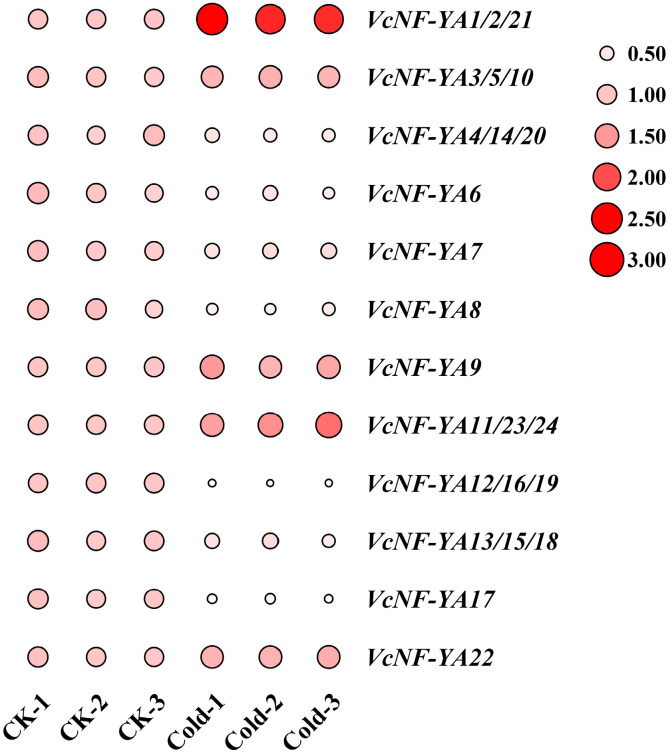
Expression analysis of *VcNF-YA*s under cold stress treatment by qRT-PCR. The concentration of salt treatment is 4 °C, and the treatment time is 0 h (CK) and 3 h. The 2^−ΔΔCt^ method was used to calculate the transcription levels of *VcNF-YA*s, and the log_2_ (sample/control) value of each *VcNF-YA* was used to show its relative expression level. Expression level indicators were laid on the right side of heatmaps. Larger size and deeper red color of a circle indicate higher relative expression level.

Cold treatment was performed for different time periods (0 h, 3 h) on blueberry seedlings, and the relative expression levels of these genes in leaves were measured by qRT-PCR (Figure 10). The results showed that *VcNF-YA1/2/21*, *VcNF-YA3/5/10*, *VcNF-YA9*, *VcNF-YA11/23/24*, and *VcNF-YA22* were upregulated, while the expression trends of *VcNF-YA4/14/20*, *VcNF-YA6*, *VcNF-YA7*, *VcNF-YA8*, *VcNF-YA12/16/19*, *VcNF-YA13/15/18*, and *VcNF-YA17* were downregulated. All the raw data of relative expression level were listed in Table S11.

### 2.10. GO Enrichmentof VcNF-YA Proteins

**Figure 11:**
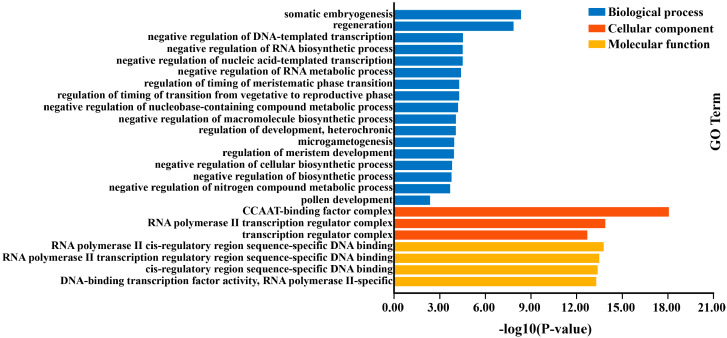
GO enrichment analysis of VcNF-YA proteins.

GO enrichment analysis was conducted to further evaluate the biological function of the VcNF-YA proteins. The result showed that VcNF-YAs were annotated with 4 molecular functional entries, 3 cellular component entries, and 17 biological process entries (Figure 11). It showed that VcNF-YAs were widely involved in various pathways and functions of plants, such as somatic embryogenesis, regeneration, and timing of meristematic phase transition. The detailed GO entries were listed in Table S13.

### 2.11. Transcription-Activating Activity of VcNF-YAs

**Figure 12:**
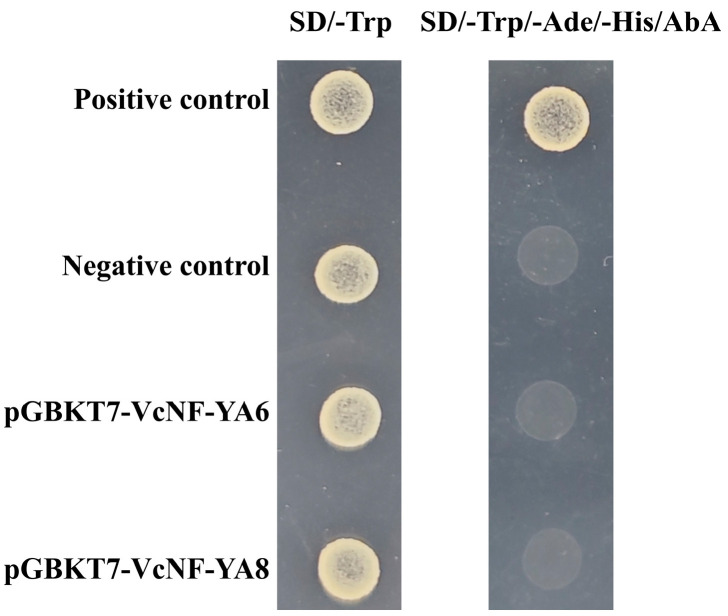
Identification of transcription-activating activity of VcNF-YA6 and VcNF-YA8. Positive control is pGBKT-53/pGADT7-T. Negative control is pGBKT7 vector. SD represents nutrition-deprived yeast culture medium. The concentration of AbA in culture medium is 500 ng/L.

*VcNF-YA6* and *VcNF-YA8*, as representatives of *VcNF-YA*s responsive to multiple abiotic stress treatments, were selected for the detection of transcription-activating activity. *VcNF-YA6* and *VcNF-YA8* genes were introduced into pGBKT7 vector and transformed into yeast cells. Neither the negative control nor the yeast transformed with pGBKT7-VcNF-YA6, and pGBKT7-VcNF-YA8 was able to grow on the nutrition-deprived culture medium added with AbA (Figure 12). It showed that VcNF-YA6 and VcNF-YA8 have no transcription-activating activity.

### 2.12. Subcellular Localization of VcNF-YA6 and VcNF-YA8 Proteins

**Figure 13:**
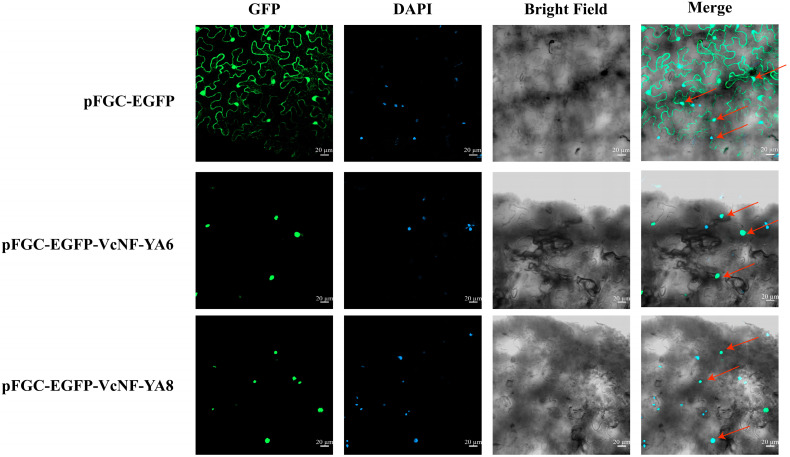
Subcellular localization of VcNF-YA6 and VcNF-YA8 proteins. DAPI staining indicates the position of the cell nucleus. Bar = 20 μm. Red arrow indicates the location of the cell nucleus in the merge field.

*VcNF-YA6* and *VcNF-YA8* genes were introduced into pFGC-eGFP vector. Transient transformation of tobacco leaf cells was performed using *Agrobacterium* containing the plasmids of pFGC-eGFP, pFGC-eGFP-NF-YA6, and pFGC-eGFP-NF-YA8, respectively. Green fluorescence distribution indicates subcellular localization. As shown in Figure 13, green fluorescence signal was able to be observed across the entire tobacco leaf cell transformed with pFGC-eGFP. In contrast, green fluorescence signal was only detected in the nucleus of tobacco leaf cells transformed with pFGC-eGFP-VcNF-YA6 and pFGC-eGFP-VcNF-YA8. It indicated that VcNF-YA6 and VcNF-YA8 proteins were localized in the nucleus. This result is consistent with the basic characteristics of nuclear localization of most transcription factors.

## 3. Discussion

*NF-Y* transcription factors are known to play crucial roles in various biological processes, including growth and development, flowering, and stress responses. Previous studies have proven that NF-YA family members are involved in the regulation of genes associated with drought resistance, seed germination, and root development in various species, such as *Arabidopsis thaliana* and *Oryza sativa* (rice) [4,22]. In this study, a total of 24 *VcNF-YA* gene family members were identified in blueberry across the whole genome. All the VcNF-YA family members contained two conserved domains, NF-YB-NF-YC interaction domain and DNA binding domain, except for VcNF-YA11, 17, 23, and 24, of which the DNA binding domain was incomplete. It indicates that the functioning of these four members may rely on protein interaction rather than gene regulation. These members were classified into four subgroups according to their phylogenetic relationships. The NF-YAs from blueberry were more likely to be classified into the same subgroup as those from kiwi fruit than from *Arabidopsis* or poplar, which may result from the closer evolutionary relationships between blueberry and kiwi fruit. Some gene combinations, such as *VcNF-YA13*, *-15*, and *-18*, *VcNF-YA3*, *-5*, *-10*, *VcNF-YA12*, *-16*, and *-19*, had similar gene structures and motif patterns, as well as collinear relationships. It indicates the high frequency of gene duplication events and function redundancy. *VcNF-YA13* showed the highest frequency in collinear gene pairs, which indicates this gene may play a more central role. The more collinear gene pairs between *VcNF-YA*s and *AcNF-YA*s also result from the closer evolutionary relationships between blueberry and kiwi fruit. *VcNF-YA* transcription factors exhibited different expression patterns in different tissues and under different stress treatments. The higher expression levels in stems and leaves compared to roots suggest that VcNF-YAs may play a more active role in stress perception. It is consistent with the findings in other plant species, where leaves and stems are often involved in early response to environmental stressors [26]. The promoters of genes such as *VcNF-YA12*, *VcNF-YA16*, and *VcNF-YA19* contain abundant MeJA-responsive elements and drought-responsive elements, and their expression levels were altered under salt and cold treatment conditions. The promoters of *VcNF-YA4*, *VcNF-YA14*, and *VcNF-YA20* genes contain abundant MeJA and ABA response elements, and they were downregulated under ABA treatment, salt treatment, and cold treatment conditions [26].

Most of the *VcNF-YA* genes were responsive to abscisic acid (ABA), salt, and cold stress, which aligns with previous research indicating that *NF-YA* factors are integral to ABA signaling pathways and stress tolerance [23]. For instance, studies in wheat have proven that *TaNF-YA* genes activated drought-responsive genes, enhancing the plant tolerance to water scarcity [30]. The analysis of *cis*-acting elements in the promoter regions of *VcNF-YA* genes revealed a rich diversity of elements associated with hormone responses and stress tolerance. The presence of ABA-responsive elements suggests that these transcription factors may function as key regulators in ABA signaling pathway, coordinating the plant response to abiotic stress. It is reasonable given the important roles of ABA in mediating drought and salt stress responses [24]. Moreover, the identification of light-responsive elements indicates that *VcNF-YA* may also integrate environmental signals such as light. This multifaceted role aligns with the concept of transcription factor “promiscuity,” where a single factor can regulate multiple pathways and responses [5]. Future research should focus on elucidating the specific target genes of VcNF-YAs and their interactions with other signaling pathways to establish a more comprehensive network of their regulatory roles.

The identification and characterization of NF-YA transcription factors in blueberry (*V. corymbosum*) promote the advancement in our understanding of how this economically important species responds to abiotic stress. The results of this study not only align with the previous research on NF-YA functions in other plant species but also highlight unique aspects of their roles in blueberry. The findings in this study have valuable implications for blueberry cultivation, particularly in the context of accelerating climate change and deteriorating abiotic stresses. Understanding the molecular mechanisms in which *VcNF-YAs* confer stress tolerance can benefit the breeding programs aimed at developing more resilient blueberry cultivars. Moreover, the identification of specific *VcNF-YA* genes that are particularly responsive to stress could serve as valuable biomarkers for selecting stress-tolerant genotypes. It can facilitate the development of cultivars that not only maintain yield under adverse conditions but also possess enhanced nutritional qualities.

In summary, this study provides valuable insights into the role of VcNF-YA transcription factors in the abiotic stress response of blueberry. The findings not only enhance our understanding of the molecular mechanisms underlying stress tolerance in blueberries but also open new avenues for research and practical applications in crop improvement. As climate change continues to pose challenges for agriculture, elucidating the genetic basis of stress resilience is essential for developing sustainable culture practices and ensuring crop quality.

## 4. Materials and Methods

### 4.1. Identification and Analysis of VcNF-YA Transcription Factor Family in Vaccinium corymbosum

The amino acid sequences of the 10 *Arabidopsis thaliana* NF-YAs (AT1G17590, AT1G30500, AT1G54160, AT1G72830, AT2G34720, AT3G05690, AT3G14020, AT3G20910, AT5G06510, and AT5G12840) were downloaded from the PlantTFDB v5.0 database (https://planttfdb.gao-lab.org/). Two methods were used to screen and identify NF-YA subfamily members in *V. corymbosum*. On the one hand, AtNF-YAs were used as query sequences to search for NF-YA sequences in *V. corymbosum* through BlastP [38]. On the other hand, the HMM file of NF-YA (CBFB_NFYA: PF02045) was retrieved from Pfam database (http://pfam.xfam.org/), and HMMER v3.1 [39] tool was used to obtain putative NF-YA family members from the *V. corymbosum* database. Additionally, the two conserved domains were used for further verification according to Tom Laloum’s method [5]. Basic characteristics of the VcNF-YA amino acid sequences were analyzed using ExPASy (http://www.expasy.org/), including molecular weight, isoelectric point, amino acid number, aliphatic index, and hydrophilic mean (GRAVY) score.

### 4.2. Analysis of Gene Structure and Conserved Motifs

The gene sequences and CDS of *VcNF-YAs* were downloaded from NCBI and then analyzed with the online software GSDS 2.0 [40] (Gene Structure Display Server: http://gsds.cbi.pku.edu.cn/) for exon-intron distribution patterns. The online software MEME 5.0 [41] (http://meme-suite.org/) was used to predict the conserved motifs of the VcNF-YA protein sequences.

### 4.3. Multiple Sequence Alignment and Phylogenetic Analysis of the 24 VcNF-YAs

Multiple sequence alignment of amino acid sequences was performed using TBtools [42] (v2.136), and the conserved motifs were checked. The phylogenetic tree (1000 bootstrap replications) was constructed with NF-YA subfamily members in *Vaccinium corymbosum*, *Populus trichocarpa*, *Actinidia chinensis*, and *Arabidopsis thaliana* with Maximum Likelihood (ML) method using TBtools (v2.136) software.

### 4.4. Chromosome Distribution, Intraspecies and Interspecies Collinearity Analysis, and Ka/Ks Calculation

TBTBtools-II (v2.136) was used for extracting chromosome location information of *VcNF-YA*s and visualization. Gff3 (General Feature Format 3) files of *A. thaiana* TAIR10, *A. chinensis* PS1, and *P. trichocarpa* v4.1 were searched and downloaded from EnsemblPlants [43] (http://plants.ensembl.org/index.html, accessed on 15 September 2024). Gff3 files of *V. corymbosum* were searched and downloaded from GDV (https://www.vaccinium.org/). TBtools software was used to construct collinear analysis diagram, explore the gene duplication events of *VcNF-YAs*, and calculate the Ka and Ks values.

### 4.5. Analysis of cis-Acting Elements

The 2000 bp sequence upstreaming of *VcNF-YA* CDS was extracted by using TBtools and was analyzed via the PlantCARE online service platform [44] (http://bioinformatics.psb.ugent.be/webtools/plantcare/html/, accessed on 28 September 2024). The generated *cis*-acting elements were visualized by TBtools.

### 4.6. Plant Materials, Growth Conditions, Treatments, and Sampling

Blueberry (*V. corymbosum*) seedlings were preserved by the School of Agriculture, Liaodong University (Dandong). The seedlings were grown for 60 days in hydroponic culture (MS medium), and then those in good and similar growth states were selected for further experimental use. For stress treatment, seedlings were treated in a hydroponic culture containing 100 μM ABA [45] or 200 mM NaCl [46]. Growth conditions were set with 16 h light/8 h darkness in a greenhouse at 25 °C. The ABA or salt treatment time was 0 h, 3 h, 6 h, 12 h, and 24 h. The blueberry seedlings were grown in tissue culture containers for 30 days before the cold treatment (4 °C, 3 h).

After ABA treatment and salt treatment, roots, stems, and leaves of the blueberry plants were sampled, respectively. After cold treatment, leaves of the blueberry plants were sampled. The nutrient solution on the tissue surface was rinsed with deionized water, and then the excess water was absorbed with absorbent tissue. The collected plant tissue samples were immediately frozen in liquid nitrogen and stored at −80 °C for subsequent analysis. In order to obtain reproducible results, there were three sets of biological replicates.

### 4.7. RNA Extraction and Expression Analysis

Total RNA was extracted from different blueberry tissues (roots, stems, and leaves) using RNA extraction kit (Bioteke, Beijing, China). RNA concentration was measured, and its quality was examined by electrophoresis with 1% agarose gel. RNA was reverse-transcribed to single-stranded cDNA using reverse transcription kit (PrimeScriptTM RT reagent Kit, Takara Bio, Kusatsu, Japan). Quantitative primers were designed according to the downloaded full-length cDNA sequences of *VcNF-YA*s, and the internal reference gene was *GAPDH* [35,47].

The cDNA obtained was diluted 10 times and used as the template for qRT-PCR. Based on SYBR Green fluorescence program, the qRT-PCR experiment was performed using THUNDERBIRD Next SYBR qPCR Mix (TOYOBO, Osaka, Japan). The total reaction volume was 20 μL. The specific reaction conditions were as follows: 95 °C for 5 min, 95 °C for 15 s, and 60 °C for 5 min for 45 cycles. The amplification reaction was carried out on Applied Biosystems 7500 Fast Real-Time PCR System (Waltham, MA, USA). All reactions were repeated three times, and the analysis of relative expression levels of genes was carried out with the 2^−ΔΔCt^ method [48].

Primers used in qRT-PCR were designed by Primer 5.0 (Premier Biosoft, Palo Alto, CA, USA), and primer specificity was tested using TBtools v2.136. Primer sequences were listed in Table S12. Due to high sequence similarity, it is of necessity that one primer pair was designed for the simultaneous quantification of a certain gene combination, which includes *VcNF-YA1/2/21*, *VcNF-YA3/5/10*, *VcNF-YA4/14/20*, *VcNF-YA11/23/24*, *VcNF-YA12/16/19*, and *VcNF-YA13/15/18*. The heatmaps of tissue-specific expression analysis of *VcNF-YA*s were constructed by using GraphPad Prism 9.0.0.

### 4.8. Identification of Transcriptional Activation Activity of VcNF-YAs

To identify the transcriptional activation activity, VcNF-YA6 and VcNF-YA8, as representatives, were introduced into pGBKT7 yeast expression vector. The recombinant pGBKT7-VcNF-YA6, pGBKT7-VcNF-YA8, pGBKT-53/pGADT7-T (positive control), and pGBKT7 (negative control) were transformed into yeast competent cells (Y2H). Afterwards, they were cultured on the nutrition-deprived yeast culture medium for 3–5 d at 30 °C. AbA (500 ng/L in culture medium) was added in the medium for the detection of whether yeast GAL4 system was activated.

### 4.9. GO Enrichment Analysis of VcNF-YA Genes

EggNOG mapper was used for GO annotation of VcNF-YA proteins. TBtools was used to visualize the results of GO enrichment analysis.

## 5. Conclusions

In this study, 24 NF-YA transcription factors were identified in the blueberry genome. They were classified into four subgroups according to their phylogenetic relationships. The members in the same subgroup had more similar exon-intron structures and motif patterns. The 24 *VcNF-YA* genes were localized on three different scaffolds. A total of 23 collinear gene pairs were identified between *VcNF-YA*s, as well as 28, 28, and 83 collinear gene pairs between blueberry and *Arabidopsis*, blueberry and poplar, and blueberry and kiwi fruit, respectively. The *cis*-acting elements in the promoter regions of *VcNF-YA*s were mainly involved in plant hormone response elements, growth and development elements, and stress response elements. Most of the *VcNF-YA* genes exhibited higher expression levels in stems and leaves compared to roots and were responsive to ABA, salt, and cold treatments. The results of GO enrichment analysis showed that the functions of VcNF-YAs were mainly enriched in somatic embryogenesis, regeneration, and timing of meristematic phase transition. Furthermore, VcNF-YA6 and VcNF-YA8 showed no transcription-activating activity and were localized in the nucleus. These results provide a reference for future research on NF-YA transcription factors and crop abiotic stress response.

## Figures and Tables

**Table 1:** Identification and physicochemical properties of the 24 NF-YA transcription factor family members in blueberry.

Gene Name	Locus Name	Amino Acid No.	Molecular Weight (Da)	Isoelectric Point	Aromaticity	Instability Index	Aliphatic Index	GRAVY
*VcNF-YA1*	Vc_DUK_00005681-RA	307	33,660.66	9.61	0.0912	61.59	61.73	−0.58
*VcNF-YA2*	Vc_DUK_00049900-RA	333	36,417.54	8.73	0.0871	59.22	60.75	−0.61
*VcNF-YA3*	Vc_DUK_00060112-RA	292	32,545.71	6.96	0.0616	66.5	51.64	−1.06
*VcNF-YA4*	Vc_DUK_00005992-RA	343	37,833.08	7.96	0.0787	50.93	61.22	−0.68
*VcNF-YA5*	Vc_DUK_00024170-RA	292	32,502.69	6.96	0.0616	67.86	51.99	−1.04
*VcNF-YA6*	Vc_DUK_00024941-RA	136	14,961.01	10.57	0.0368	74.77	69.71	−0.74
*VcNF-YA7*	Vc_DUK_00022255-RA	207	23,013.43	6.41	0.0676	64.11	52.42	−1.01
*VcNF-YA8*	Vc_DUK_00052734-RA	283	31,469.02	9.06	0.0459	57.26	55.51	−1.02
*VcNF-YA9*	Vc_DUK_00014736-RA	210	23,137.95	9.33	0.0667	74.77	57.76	−0.81
*VcNF-YA10*	Vc_DUK_00015745-RA	292	32,589.83	6.96	0.0616	65.48	51.64	−1.05
*VcNF-YA11*	Vc_DUK_00044442-RA	130	14,694.45	9.75	0.1077	39.08	105.85	0.06
*VcNF-YA12*	Vc_DUK_00011698-RA	330	35,606	10.2	0.0727	58.77	60	−0.59
*VcNF-YA13*	Vc_DUK_00013377-RA	332	36,651.14	8.9	0.0602	61.1	60.54	−0.73
*VcNF-YA14*	Vc_DUK_00034638-RA	305	33,701.71	9.43	0.0721	48.39	63.05	−0.67
*VcNF-YA15*	Vc_DUK_00025714-RA	332	36,651.14	8.9	0.0602	61.1	60.54	−0.73
*VcNF-YA16*	Vc_DUK_00027345-RA	330	35,814.3	10.26	0.0727	59.37	60.27	−0.61
*VcNF-YA17*	Vc_DUK_00036894-RA	110	12,382.36	9	0.0636	64.42	86.09	−0.36
*VcNF-YA18*	Vc_DUK_00060457-RA	332	36,557.11	9.27	0.0633	59.98	59.1	−0.73
*VcNF-YA19*	Vc_DUK_00061817-RA	330	35,773.25	10.2	0.0727	57.96	60.85	−0.59
*VcNF-YA20*	Vc_DUK_00070132-RA	343	37,763	7.91	0.0787	52.89	60.09	−0.67
*VcNF-YA21*	Vc_DUK_00039037-RA	319	35,103.18	9.52	0.0878	62.31	60.34	−0.65
*VcNF-YA22*	Vc_DUK_00094119-RA	208	22,994.42	6.41	0.0625	65.96	55.91	−0.94
*VcNF-YA23*	Vc_DUK_00099783-RA	106	12,033.23	9.76	0.0755	65.2	95.85	−0.3
*VcNF-YA24*	Vc_DUK_00101891-RA	115	12,983.33	9.77	0.0957	66.15	90.87	−0.29

## Data Availability

The original contributions presented in this study are included in the article and Supplementary Materials. Further inquiries can be directed to the corresponding authors.

## Supplementary Materials

The following supporting information can be downloaded at: https://www.mdpi.com/article/10.3390/ijms252312832/s1.
